# A Therapeutic Peptide Vaccine Against PCSK9

**DOI:** 10.1038/s41598-017-13069-w

**Published:** 2017-10-02

**Authors:** Yajie Pan, Yanzhao Zhou, Hailang Wu, Xiao Chen, Xiajun Hu, Hongrong Zhang, Zihua Zhou, Zhihua Qiu, Yuhua Liao

**Affiliations:** 1 Department of Cardiology, Union Hospital, Tongji Medical College, Huazhong University of Science and Technology, Wuhan, 430022 China; 2 Institute of Cardiology, Union Hospital, Tongji Medical College, Huazhong University of Science and Technology, Wuhan, 430022 China; 3 Key Lab of Molecular Biological Targeted Therapies of the Ministry of Education, Union Hospital, Tongji Medical College, Huazhong University of Science and Technology, Wuhan, 430022 China

## Abstract

Vaccination provides a promising approach for treatment of hypercholesterolemia and improvement in compliance. In this study, the appropriate virus-like particle (VLP)-peptide vaccines targeting proprotein convertase subtilisin/kexin type 9 (PCSK9) were screened. The screening criteria of target peptides were as follows: (1) located in catalytic domain of PCSK9, or regulating the binding of PCSK9 and LDL receptors (LDLR); (2) having low/no-similarity when matched with the host proteome; (3) possessing ideal antigenicity and hydrophilicity; (4) including the functional mutation site of PCSK9. It was found that mice vaccinated with VLP -PCSK9 peptide vaccines, especially PCSK9Qβ-003 vaccine, developed high titer IgG antibodies against PCSK9. PCSK9Qβ-003 vaccine obviously decreased plasma total cholesterol in both Balb/c mice and LDLR^+/−^ mice. Also, PCSK9Qβ-003 vaccine decreased plasma PCSK9 level and up-regulated LDLR expression in liver. Additionally, PCSK9Qβ-003 vaccine injection was associated with significant up-regulation of sterol-regulatory element-binding protein-2 (SREBP-2), hepatocyte nuclear factor 1α (HNF-1α), and 3-hydroxy-3-methylglutaryl coenzyme A (HMG-CoA) reductase in LDLR^+/−^ mice. No obvious immune injury was detected in vaccinated animals. The PCSK9Qβ-003 vaccine, therefore, may be an attractive treatment approach for hypercholesterolemia through decreasing cholesterol and regulating lipid homeostasis.

## Introduction

Increase in low-density lipoprotein cholesterol (LDL-C) is a major risk of atherosclerosis and ischemic cardiovascular diseases (CVD). Statin can significantly reduce LDL-C, and is the most commonly used drug to treat hypercholesterolemia^1^. However, intensive statin therapy still has residual risks and 20% of high-risk patients with hypercholesterolemia could not achieve adequate control of LDL-C^2,3^. Plasma LDL-C is removed from circulation when it interacts with LDL receptors (LDLR) which are abundant on hepatocytes in liver^4^. Upon LDLR binding, LDL-C is endocytosed and undergoes lysosomal catabolism in hepatocytes. Then LDLR is recycled back to the hepatocytes surface. Proprotein convertase subtilisin/kexin type 9 (PCSK9) is a hepatic enzymatic protein that negatively regulates LDLR. Plasma PCSK9 binds to the extracellular domain of LDLR, and then mediates internalization and degradation of LDLR, which results in the increase of LDL-C level. Genetic studies have shown that gain-of-function mutations in PCSK9 are associated with autosomal dominant hypercholesterolemia^5^, while loss-of-function mutations are associated with increase in the LDLR surface expression and increased levels of LDL internalization^6^.

To date, the most advanced approach for PCSK9 inhibition is monoclonal antibody (mAb). The famous alirocumab and evolocumab were approved by FDA in 2015. Although shown to lower LDL-C significantly, the use of mAb faces functional limitations because of frequent administration and high costs. Active vaccination approach could circumvent these drawbacks. Display of self-antigens in a highly dense, repetitive format on the surface of virus-like particles (VLPs) is one approach for inducing strong antibody responses against self-antigens^7,8^. VLP display has been successfully used to target self molecules that are involved in the pathogenesis of a variety of chronic diseases. Clinical trials showed that VLP-based angiotensin II vaccine (CYT-006-AngQβ) was highly immunogenic and significantly reduced blood pressure in hypertensive patients^9^. Our team have invented a VLP-based anti-hypertensive vaccine against human and murine angiotensin II receptor type 1 (ATRQβ-001), which could significantly reduce the blood pressure and protect target organs of hypertensive animals, even ameliorate atherosclerosis and nephropathy in animal models^10–12^.

In this study, given the important role of PCSK9 in regulating LDL-C metabolism, we screened and identified a Qβ bacteriophage VLP-peptide vaccine (designated PCSK9Qβ-003 vaccine) that elicits strong antibody responses against PCSK9. PCSK9Qβ-003 vaccine obviously decreased total cholesterol (TC) and up-regulated LDLR expression in both Balb/c mice and LDLR^+/−^ mice. And, PCSK9Qβ-003 vaccine was associated with significant up-regulation of sterol-regulatory element-binding protein-2 (SREBP-2), hepatocyte nuclear factor 1α (HNF-1α), and 3-hydroxy-3-methylglutaryl coenzyme A (HMG-CoA) reductase in LDLR^+/−^ mice.

## Results

### Selection and screening of the appropriate PCSK9 peptides vaccine

**Figure 1:**
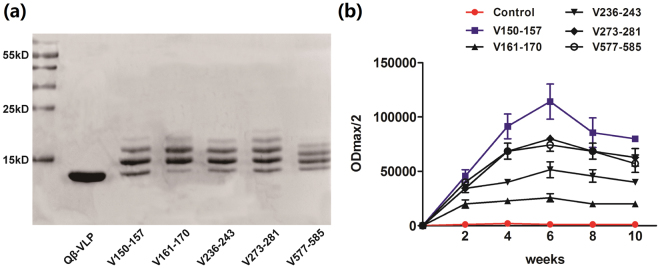
Selection and identification of the appropriate PCSK9 peptides vaccine. (**a**) The vaccine was analyzed on a SDS-PAGE gel. The figure showed the PCSK9 peptides conjugated to the VLP(full-length gel is presented in Supplementary Figure 1). (**b**) The Balb/c mice were immunized subcutaneously on days 0, 14, 28, and 56. The antibody titers were measured by ELISA as ODmax/2 on days 14, 28, 42, 56 and 70.

According to the structure and amino acid sequence of human PCSK9, 5 B cell epitope peptides were selected^13^. The peptides were conjugated with Qβ VLP, and the conjugation rate of PCSK9Qβ-003 vaccine was determined by SDS-PAGE, which manifested that one monomer of VLP could couple with one to four PCSK9 epitopes (two PCSK9 epitope per one VLP monomer averagely, Fig. 1a). Male Balb/c mice were vaccinated on days 0, 14, 28, and 56. ELISA confirmed that the anti-PCSK9 peptide antibody titer was 1:20,000~1:120,000. Especially peptide V150-157 (termed PCSK9Qβ-003 vaccine), the antibody titer of which was 1:80,000~1:120,000 after the second immunization (Fig. 1b). These indicated that the selected peptides had high antigenicity.

**Figure 2:**
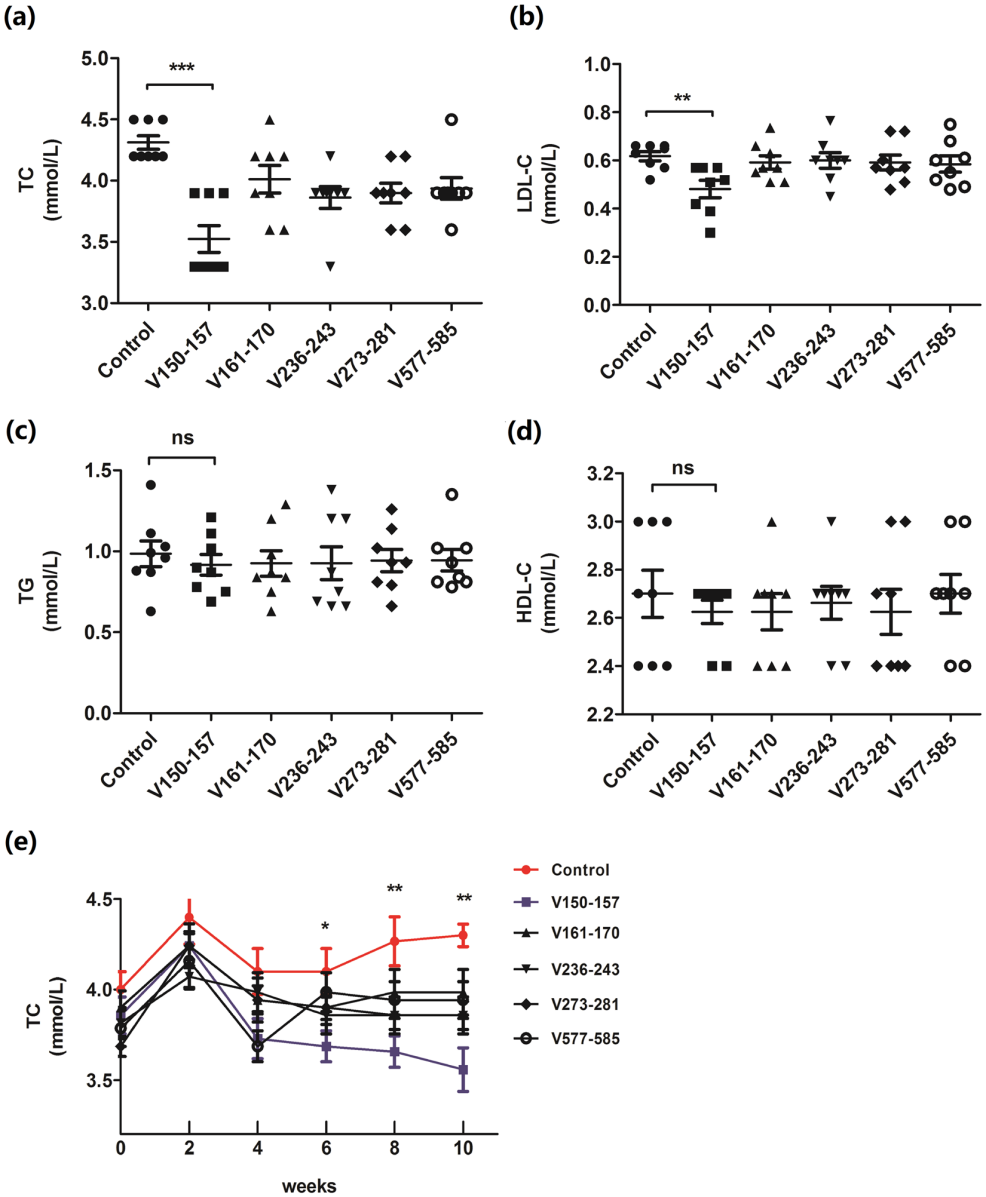
Screening of the appropriate PCSK9 peptides vaccine. Compared to the control group, significant reduction in TC and LDL-C following PCSK9Qβ-003 vaccination was observed in Balb/c mice, while there was no significant difference in other vaccine groups. (**a**) TC was decreased in the PCSK9Qβ-003 vaccine group compared with the control group. (**b**) LDL-C level in the PCSK9Qβ-003 vaccine group was significantly decreased. (**c**,**d**) No significant difference of TG and HDL-C level was observed among all groups. (**e**) PCSK9Qβ-003 vaccine reduced TC in Balb/c mice since day 42 to the end of the study. n = 8 per group. Data are expressed as means ± SEM. *P < 0.05, **P < 0.01,***P<0.001 vs. the control group. ns: no significant difference.

To evaluate the functional effect of immunization against the various PCSK9 epitopes, the lipids level was detected in Balb/c mice. It was showed that, compared to the control group, significant decrease in TC and LDL-C following PCSK9Qβ-003 vaccination was observed in Balb/c mice after the third injection, while other PCSK9 peptides vaccines had no prominent influence on plasma cholesterol (Fig. 2). The TC was decreased by 20% after the fourth vaccination in PCSK9Qβ-003 vaccine group (Fig. 2a). No significant difference of triglyceride (TG) and high-density lipoprotein cholesterol (HDL-C) level was observed among groups (Fig. 2c,d). As a result, PCSK9Qβ-003 vaccine may be a candidate vaccine for treating hypercholesterolemia.

### PCSK9Qβ-003 vaccine decreased TC in LDLR^+/−^ mice

**Figure 3:**
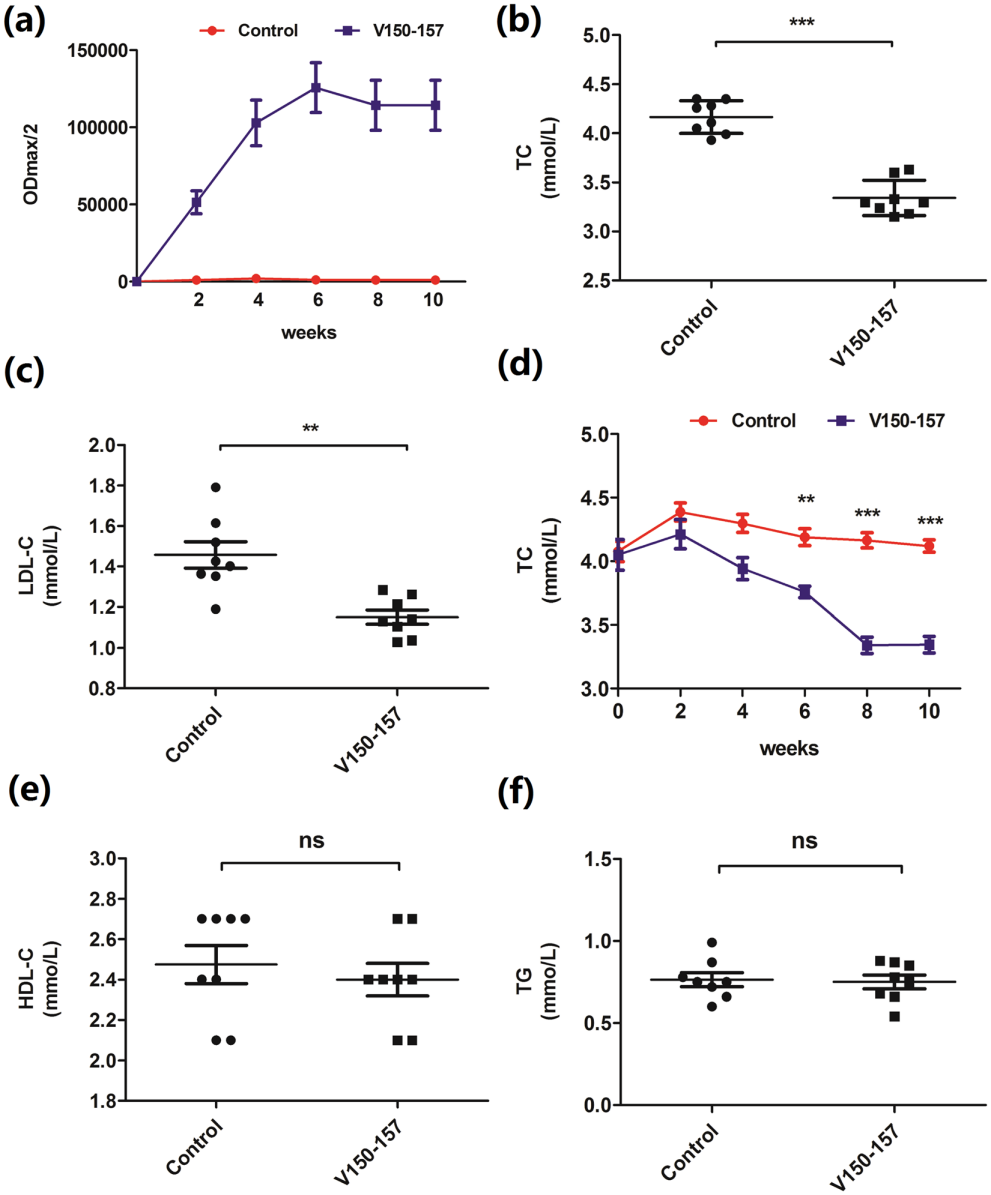
PCSK9Qβ-003 vaccine decreased TC and LDL-C in LDLR^+/−^ mice. (**a**) PCSK9Qβ-003 vaccine elicited strong antibody production against PCSK9-003 peptide after the second immunization in LDLR^+/−^ mice. (**b**,**c**) Compared with the control group, TC and LDL-C level was decreased in the PCSK9Qβ-003 vaccine group. (**d**) PCSK9Qβ-003 vaccine lowered TC level from day 42 to the end of the study. (**e**,**f**) There was no significant difference of HDL-C and TG between the two groups. n = 8 per group. Data are expressed as means ± SEM. *P < 0.05, **P < 0.01, ***P < 0.001 vs. the control group. ns: no significant difference.

To confirm the effect of PCSK9Qβ-003 vaccine on hypercholesterolemia, PCSK9Qβ-003 vaccine was used to vaccinate male LDLR^+/−^ mice. Similarly, PCSK9Qβ-003 vaccine elicited high titer antibody production (1:80,000~1:160,000) against PCSK9-003 peptide after the second immunization (Fig. 3a). Obvious decline in cholesterol and LDL-C following PCSK9Qβ-003 vaccination was observed in LDLR^+/−^ mice after the third injection, compared with the control group (15–25% decrease; Fig. 3b,c). And this effect continued until the end of the study (Fig. 3d). HDL-C and TG levels were not affected in the vaccine group (Fig. 3e,f). No significant difference of body weight, blood pressure and heart rate was observed in both the control and the vaccine groups (Supplementary Table S1).

**Figure 4:**
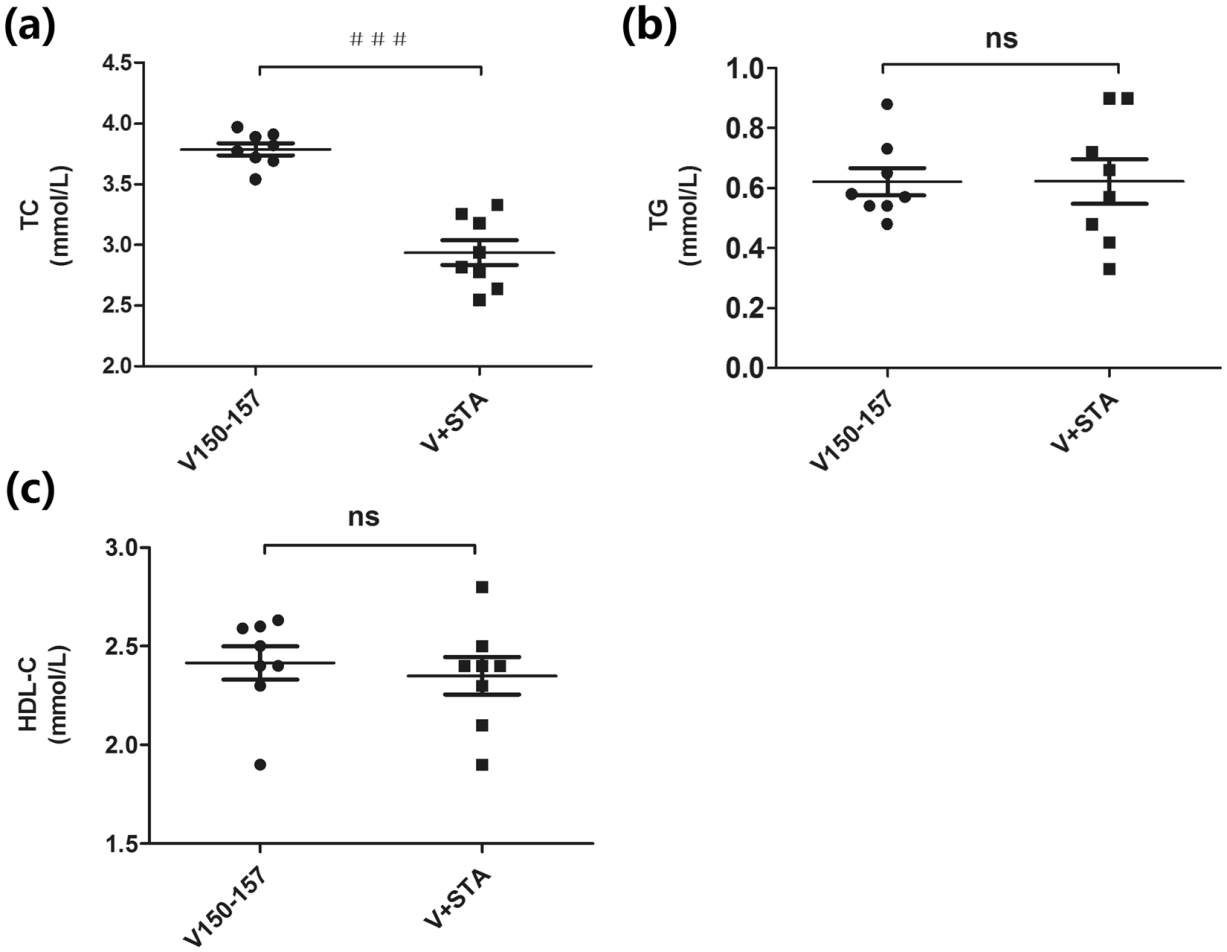
The synergistic effect of PCSK9Qβ-003 vaccine with statin. (**a**) Compared to the PCSK9Qβ-003 vaccine group, treatment with statin in the vaccinated mice further reduced TC by 20%. (**b**,**c**) No significant difference was observed in sera concentration of TG and HDL-C between the two groups. n = 8 per group. Data are expressed as means ± SEM. ^###^P < 0.001 vs. the PCSK9Qβ-003 vaccine group. ns: no significant difference.

Further, to assess whether vaccination worked synergistically with statin, simvastatin (10 mg/kg/day) was introduced into the immunized mice for 4 weeks on day 70. The plasma lipids were re-measured on day 98. Vaccinated mice treated with simvastatin had a dramatic and statistically significant further 20% reduction in TC compared to the control mice (Fig. 4a). TG and HDL-C levels were virtually unaffected in both groups (Fig. 4b,c). The results showed that the lipids were decreased more under the combined treatment of statin with PCSK9Qβ-003 vaccine.

### PCSK9Qβ-003 vaccine decreased the level of free PCSK9

**Figure 5:**
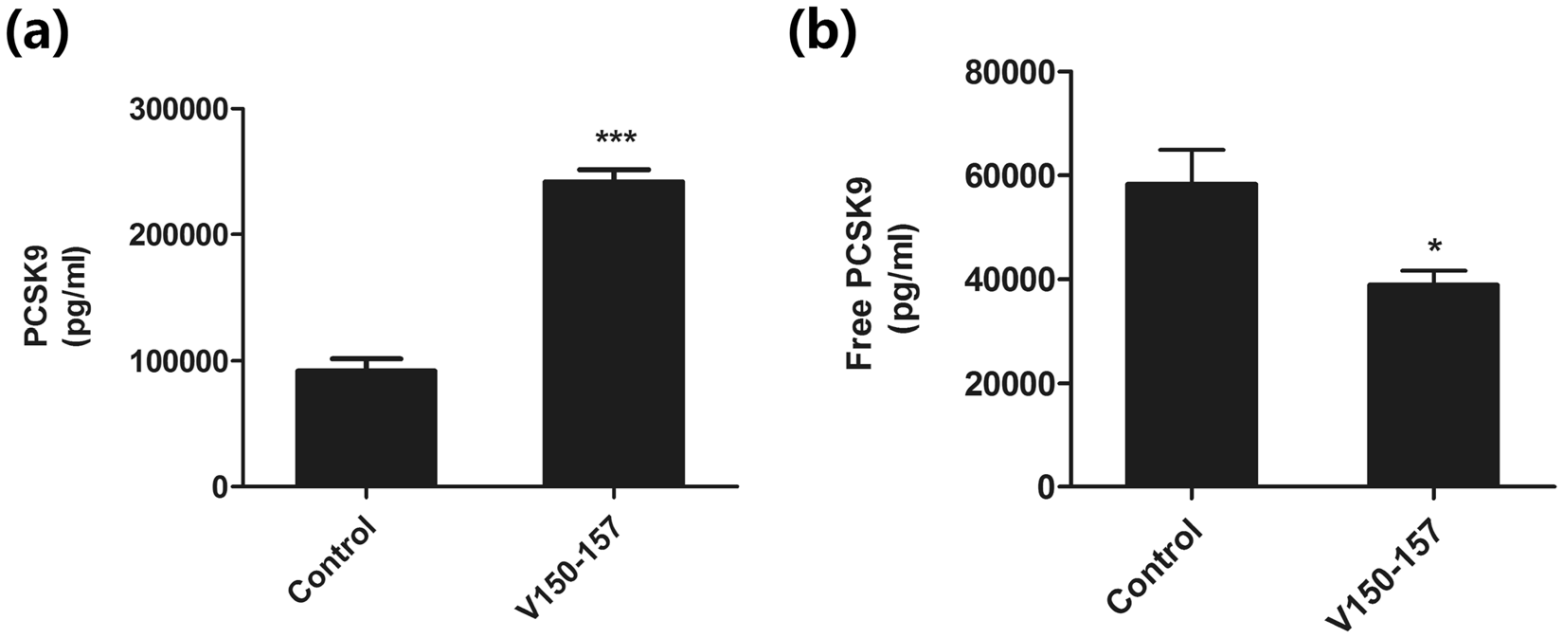
PCSK9Qβ-003 vaccine decreased the level of free PCSK9 in LDLR^+/−^ mice. (**a**) The total PCSK9 level was significantly elevated in the PCSK9Qβ-003 vaccine group compared to the control mice. (**b**) The free PCSK9 level was substantially decreased in the PCSK9Qβ-003 vaccine group compared with the control mice. n = 8 per group. Data are expressed as means ± SEM. *P < 0.05, ***P < 0.001 vs. the control group.

To evaluate plasma PCSK9 level, PCSK9 level was measured before vaccination and after the fourth immunization with PCSK9Qβ-003 vaccine. Total PCSK9 level was significantly elevated in mice that had been vaccinated with PCSK9Qβ-003 vaccine compared to the control mice (Fig. 5a). In clinical trial, anti-PCSK9 mAbs actually raise plasma PCSK9 level because of that anti-PCSK9 antibodies engage PCSK9 *in vivo* and form an immune complex^14^. The increased detection of total PCSK9 in the vaccine group may be largely due to the presence of immunoglobulin-bound PCSK9. To exclude the effect of anti-PCSK9 antibodies on total soluble PCSK9 level in the plasma, plasma was treated with magnetic Protein G-coupled beads to remove immune complexes. It was showed that free PCSK9 level was substantially decreased by about 30%, similar to anti-PCSK9 mAbs (Fig. 5b). Also, these data provided evidence that IgG elicited by vaccination binds to PCSK9 *in vivo*.

### PCSK9Qβ-003 vaccine regulated lipid homeostasis

**Figure 6:**
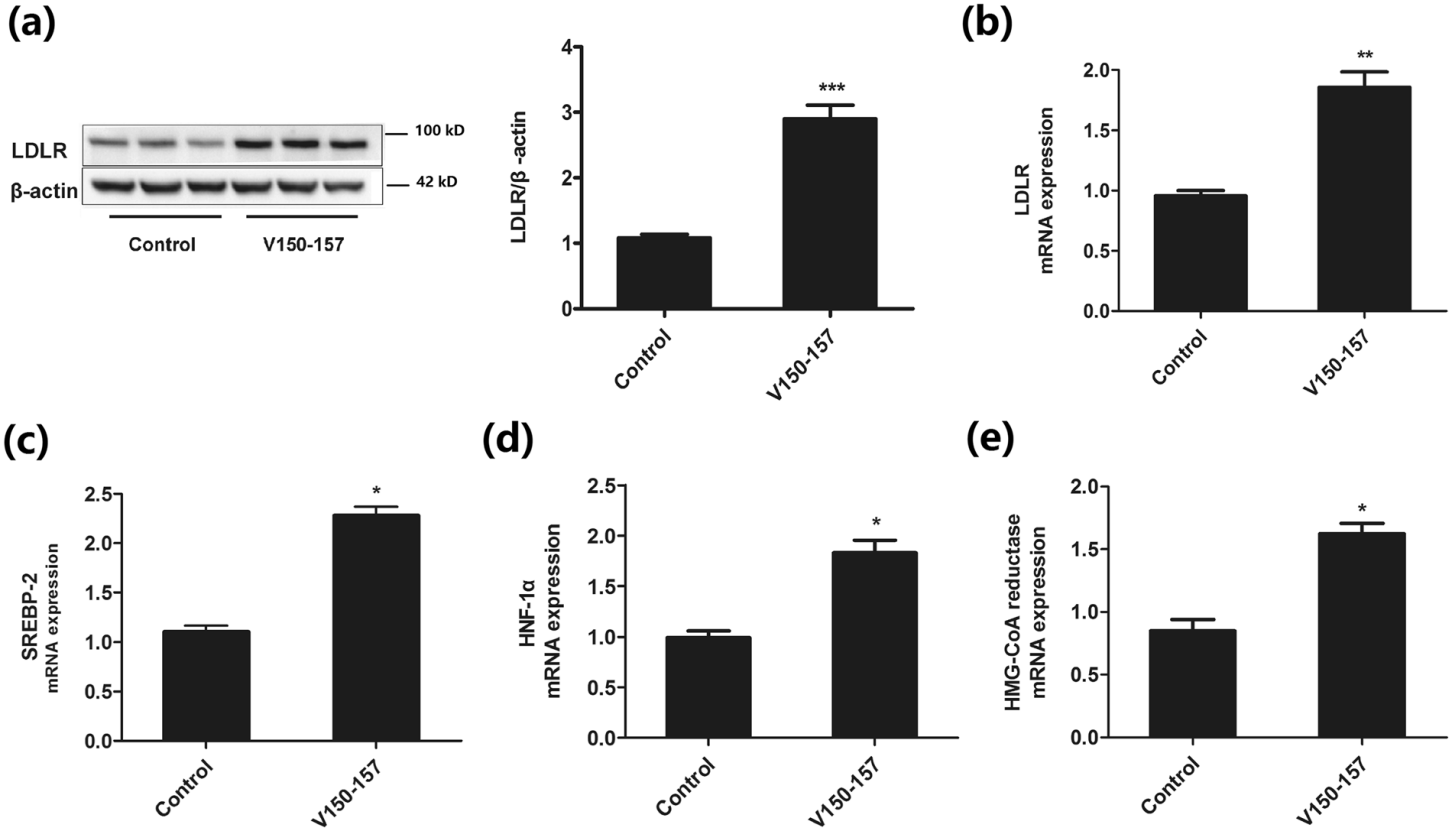
PCSK9Qβ-003 vaccine up-regulated LDLR expression and regulated lipid homeostasis. (**a**,**b**) Compared to the control group, the level of LDLR mRNA and protein expression in liver was increased obviously in the PCSK9Qβ-003 vaccine group, the samples derive from the same experiment and that blots were processed in parallel (the primary blots are presented in Supplementary Figure 2, 3). (**c**,**d**,**e**) PCSK9Qβ-003 vaccine up-regulated the mRNA level of SREBP-2, HNF-1α and HMG-CoA reductase. n = 8 per group. Data are expressed as means ± SEM. *P < 0.05, **P<0.01, ***P < 0.001 vs. the control group.

The antibody induced by PCSK9Qβ-003 vaccine lowered LDL-C through blocking PCSK9 activity, which degraded LDLR of hepatocyte. To determine the LDLR level, the liver LDLR was detected by qRT-PCR and western blot. Compared to the control group, the level of LDLR mRNA and protein expression in liver was increased obviously in the PCSK9Qβ-003 vaccine group in LDLR^+/−^ mice (Fig. 6a,b). The same result was also observed in the vaccinated Balb/c mice. These findings suggested that PCSK9Qβ-003 vaccine could inhibit the degradation of LDLR induced by PCSK9.

Statin therapy up-regulates the transcription factor SREBP-2, which activates the Ldlr and Pcsk9 genes, and thus makes statin somewhat self-limiting to further reduce LDL-C^15^. To assess the effect of PCSK9Qβ-003 vaccine on lipid homeostasis in LDLR^+/−^ mice, we evaluated the mRNA expression of SREBP-2, HNF-1α and HMG-CoA reductase in liver. Similarly, compared to the control group, the result indicated that PCSK9Qβ-003 vaccination up-regulated the level of the transcription factors SREBP-2 and HNF-1α (Fig. 6c,d). And the mRNA expression of HMG-CoA reductase was also increased in the PCSK9Qβ-003 vaccine group (Fig. 6e).

### No obvious injury was observed in the vaccinated animals

**Figure 7:**
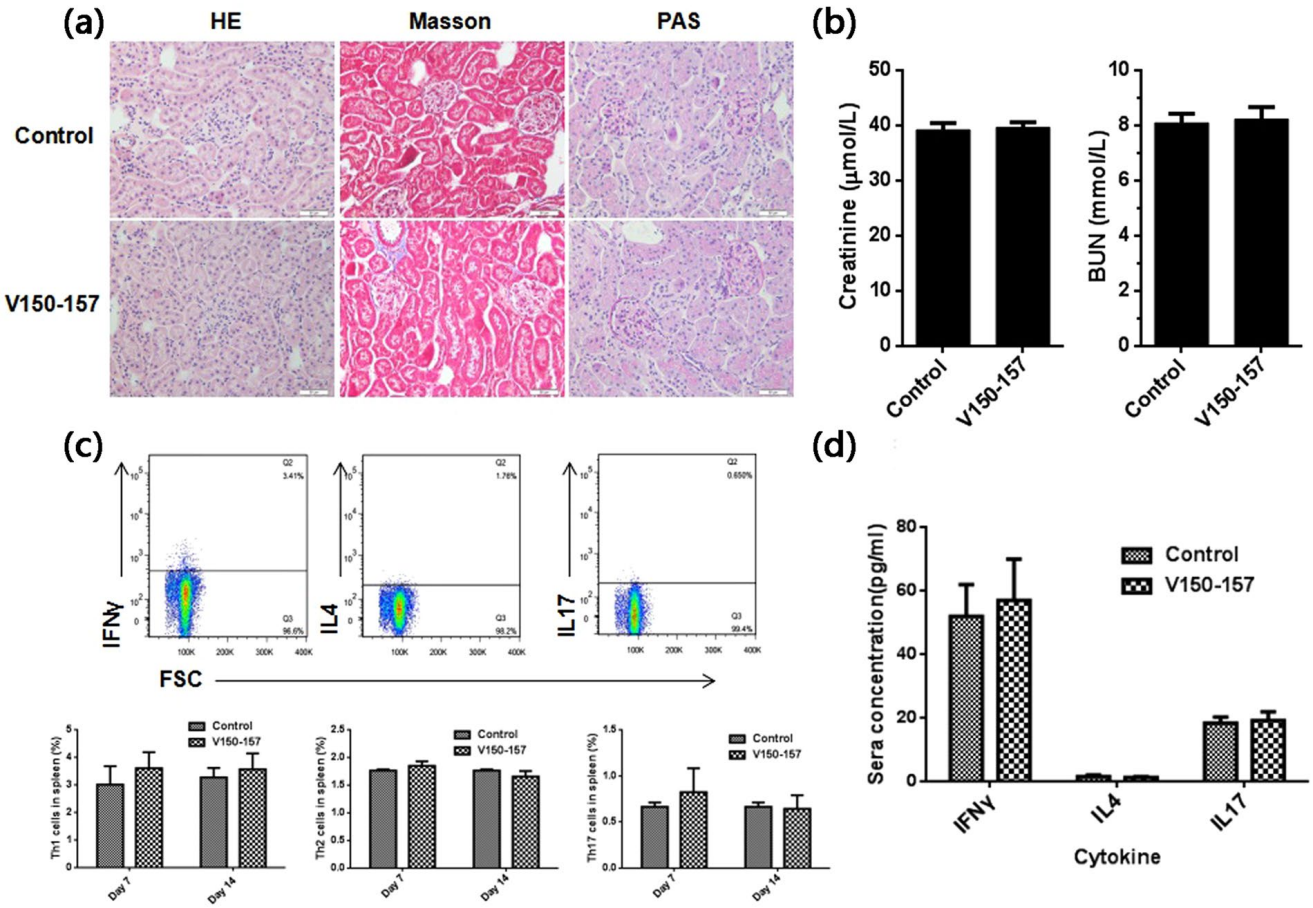
No immune-mediated injury was observed in vaccinated animals. (**a**) No significant damage was detected in vaccinated animals through HE, Masson and PAS staining. (**b**) The renal function including sera creatinine and urea nitrogen (BUN) had no difference between the control group and the PCSK9Qβ-003 vaccine group. (**c**) No Th1/Th2/Th17 biased differentiation was observed in immunized mice. (**d**) The level of IFNγ, IL4 and IL17 in sera showed no difference between the PCSK9Qβ-003 vaccine group and the control group. n = 6 per group. Data are expressed as means ± SEM.

For safety consideration, the kidney histological changes of LDLR^+/−^ mice were observed through hematoxylin-eosin (HE), Masson’s trichrome (Masson) and periodic acid-schiff (PAS) staining. Results showed, no significant damage was detected in the vaccinated animals (Fig. 7a). The renal function including sera creatinine and urea nitrogen had no difference between the control group and the PCSK9Qβ-003 vaccine group (Fig. 7b). Further, the spleen of normal Balb/c mice injected with PCSK9Qβ-003 vaccine was analysed to detect the trend of T helper (Th) cells differentiation. It was showed that, compared to the control group, no Th1/Th2/Th17 biased differentiation was observed in the vaccinated group (Fig. 7c). The concentration of cytokines IFNγ, IL4 and IL17 in sera showed no difference between the two groups (Fig. 7d).

## Discussion

PCSK9 has become one of the most promising therapeutic targets for hypercholesterolemia and ischemic CVD. The most famous example is PCSK9-specific mAb, including evolocumab (Amgen) and alirocumab (Aventis/Regeneron). They could work synergistically with statin, markedly lower LDL-C level by about 60%, and reduce the incidence of cardiovascular events in clinical trials^16–18^. Nevertheless, mAbs also face important shortcomings and limitations because of frequent administration, anti-mAb antibodies production, high doses and high costs. In contrast, active vaccination provides a promising approach for treatment of hypercholesterolemia and could over the shortcomings^7,19,20^. The development of a vaccine that actively immunizes patients against PCSK9 and effectively and safely lowers LDL-C would be highly desirable.

To develop an active vaccination strategy targeting PCSK9, we displayed linear epitopes that were exposed on the surface of the molecule in proximity to the LDLR binding site on the surface of bacteriophage VLPs. Though the crystal structure of many protein antigens has been resolved, the definition of the antigenic determinants of a protein is not clearly established. Universally, the location of epitopes on the surface of the molecule, their hydrophilicity, and their segmental mobility should be evaluated. PCSK9 belongs to the subtilisin family of serine proteases and consists of a prodomain, catalytic domain, and C-terminal V domain. PCSK9 modulates LDL-C levels by a direct interaction between repeat A of the LDLR EGF homology domain and the PCSK9 catalytic domain^4,13^. The prodomain remains noncovalently associated with the catalytic domain and seems to play an important role in the catalytic activity transformation of PCSK9. The C-terminal V domain of PCSK9 participates in regulating the binding of LDLR and PCSK9. Gain-of-function mutations in PCSK9 are associated with hypercholesterolemia^5^, while loss-of-function mutations are associated with increase of LDL internalization^6^. Based on the above information, 5 potential epitopes were designed. Moreover, Kanduc and colleagues present the low-similarity hypothesis, which defines the immune unit as a sequence with no/low similarity to the host proteome and supporting the concept that immunogenicity is preferentially associated to no/low-similarity sequences^21,22^. No/low similarity peptides mean effectiveness without the risk of autoimmune phenomena. We adopted this hypothesis for peptide selection in this investigation. All our selected peptides showed excellent immunogenicity and elicited strong antibody responses. And, it was found that PCSK9Qβ-003 vaccine obviously decreased TC and LDL-C in both Balb/c mice and LDLR^+/−^ mice. PCSK9Qβ-003 vaccine decreased free PCSK9 level and up-regulated LDLR expression in LDLR^+/−^ mice. All these results revealed that PCSK9Qβ-003 vaccine may be a candidate vaccine for the treatment of hypercholesterolemia.

Statin therapy alone inhibits the activity of HMG-CoA reductase and increases circulating levels of PCSK9 by as much as 30%, compared with placebo, making them somewhat self-limiting to further reduce LDL-C^15^. This likely occurs because the transcription factor SREBP-2, that is indirectly up-regulated by statin, activates the Ldlr and Pcsk9 genes^23,24^. Vaccination decreased PCSK9 level and up-regulated the level of SREBP-2, HNF-1α and HMG-CoA reductase, which indicated that PCSK9Qβ-003 vaccine also regulated lipid homeostasis. The effect of combination application of PCSK9 vaccine and statin may be better in high-risk patients. Our results and clinical trials had indicated that PCSK9 vaccination or mAb worked synergistically with statin^18^. In general, active immunization triggers moderate change, just like the manifestation of our PCSK9Qβ-003 vaccine, while mAb induces prominent alteration. Therefore, PCSK9Qβ-003 vaccine may be more suitable to prevent and treat CVD in combination with statin or in vulnerable populations that are either resistant to statin therapy or statin intolerant.

To our knowledge, two other groups (AFFiRiS AG and Erin Crossey *et al*.) are pursuing an active vaccination approach targeting PCSK9. The peptide sequence of AFFiRiS is homologous, but not identical, to region in PCSK9 (the original sequence PCSK9 153-162)^25^. The peptide is linked to a KLH-carrier, and has generated interesting data in animals. Erin Crossey *et al*. generated a VLP-based vaccines targeting PCSK9 (the original sequence PCSK9 207-223)^26^. Mice and macaques vaccinated with the vaccine developed high titer IgGs which bound to circulating PCSK9. And the vaccination was associated with significant reductions in TC, free cholesterol, phospholipids, and TG in primates. In our previous work, the peptide (PCSK9 153-162) was also selected, whereas just slight decrease of cholesterol was detected. The difference is that the PCSK9-003 peptide (V150-157) spans prodomain and catalytic domain of PCSK9 protein. And, indeed, PCSK9Qβ-003 vaccine obviously decreased TC and LDL-C in both Balb/c mice and LDLR^+/−^ mice. PCSK9Qβ-003 vaccine may influence the catalytic activity transformation and catalytic activity at the same time.

The safety of PCSK9Qβ-003 vaccine is an important concern. The target peptide of the vaccine was B cell epitope and 8 amino acids only in length. It is not easy to induce a T cell response^10,20^. Peptide-VLP hypertension vaccine (CYT-006-AngQβ) showed good safety in clinical trials^9^. A similar peptide-VLP hypertension vaccine ATRQβ-001 invented by our group also showed no obvious inflammation lesions in important organs, which indicated that peptide-VLP vaccine is safe for the immunized animals^10–12^. The two PCSK9 vaccines mentioned above did not cause immune injury in important organs and tissues of animals^25,26^. More importantly, we have done the safety study on PCSK9Qβ-003 vaccine. The results showed that no potential T cells, particularly Th1 and Th17 cell-mediated immune-pathology was observed. PCSK9Qβ-003 vaccination did not cause immune injury in kidney of mice. No obvious renal function impairment was observed in the vaccine group. These results indicated PCSK9Qβ-003 vaccine may have good safety. Nevertheless, the potential toxicity of PCSK9Qβ-003 vaccine needs to be further investigated.

In summary, PCSK9Qβ-003 vaccine can effectively decrease lipid levels and work synergistically with statin in mice. Thus, PCSK9Qβ-003 vaccine could serve as a cost-effective supplementary to prevent and treat CVD in combination with statin or in vulnerable populations that are either resistant to statin therapy or statin intolerant.

## Materials and Methods

### Ethics statement

The study was carried out in strict accordance with the Guidelines for the Care and Use of Laboratory Animals (Science & Technology Department of Huibei Province, PR China, 2005). The protocol was approved by Animal Care and Use Committee of Hubei Province (No: 00009367, 00021468). Mice were housed under specific pathogen-free conditions with 12:12 light:dark cycle and 22 ± 2 °C and 60 ± 5 °C humidity. Sterile water and chow were available ad libitum. All efforts were made to minimize suffering and the procedure was performed under sodium pentobarbital anesthesia if neccessary.

### Peptides synthesis and vaccines preparation

According to the structure and sequence of human PCSK9^13^, 5 B cell epitopes peptides (V150-157, V161-170, V236-243, V273-281, and V577-585) as belonging to potential epitopes of human PCSK9 had been selected (Table 1). The main criteria for their choice were based on: (1) the peptides either are located in catalytic domain of PCSK9, or regulate the binding of PCSK9 and LDLR; (2) matched with the host proteome, the peptides have low/no-similarity; (3) the peptides have ideal antigenicity and hydrophilicity; (4) the peptides include the functional mutation site of PCSK9. Besides, the amino acid number of each selected peptide should be not more than 10. The peptides were synthesized by GL Ltd. (Shanghai, China) and the purity was above 98%. The PCSK9 peptides were covalently conjugated with Qβ VLP by cross-linker Sulfo-SMCC (Pierce) according to the manufacturer’s instruction. The vaccine concentration was determined using Bradford protein assay kit (Pierce).

**Table 1:** Peptides sequence.

Protein position	Sequence	Domain
V150-157	FAQSIPWN	Pro-domain, Catalytic domain
V161-170	ITPPRYRADE	Catalytic domain
V236-243	GRDAGVAK	Catalytic domain
V273-281	KSQLVQPVG	Catalytic domain
V577-585	PVLRPRGQP	C-terminal domain

### Animals vaccination

Specific Pathogen-Free (SPF grade) male Balb/c mice, weighing 20 g, aged 6 weeks, were purchased from Beijing HFK Bio-Technology Co., LTD, and fed in a specific pathogen-free environment in Laboratory Animal Center, Tongji Medical College, Huazhong University of Science and Technology. The mice were randomly divided into 1 control group and 5 PCSK9 peptides vaccine groups (n = 8). The vaccine groups were immunized subcutaneously (sc.) on days 0, 14, 28, and 56 with 100 μg PCSK9 peptides vaccine formulated in aluminum hydroxide gel respectively. The control group was given normal saline injection correspondingly ad. The vaccine specific peptides antibody titers were detected on days 0, 14, 28, 42, 56 and 70 by ELISA. All mice were sacrificed on day 70.

In addition, normal male BALB/c mice (n = 12) were divided into two groups and immunized with PCSK9Qβ-003 vaccine (100 μg) or normal saline respectively. Mice were sacrificed on days 7 and 14 after immunization. Spleen was separated and cell suspensions was prepared by mechanical trituration. Fitc-conjugated anti-CD4 antibody, PE-conjugated anti-IFNγ antibody, APC-conjugated anti-IL4 antibody, and PE/Cy7-conjugated anti-IL17 antibody were purchased from ebioscience. The ratio of Th1 cell, Th2 cell and Th17 cell in the spleen was measured by BD LSRII flow cytometer system (BD Biosciences). The concentration of IFNγ, IL4 and IL17 in sera was detected by ELISA kit (Neobioscience).

LDLR^−/−^ mice (B6.129S7-Ldlrtm1Her/L) were purchased from the Jackson laboratory (Bar Harbor, ME). Wild type (WT) C57BL/6 mice were from Beijing HFK Bio-Technology Co., LTD. All animals were fed in a same environment like Balb/c mice. LDLR^+/−^ mice were bred using F1 from WT and LDLR^−/−^ mice. The mice were randomly divided into the control group and the PCSK9Qβ-003 vaccine group (n = 8). The vaccine group was immunized sc. on days 0, 14, 28, and 56 with 100 μg PCSK9Qβ-003 vaccine formulated in aluminum hydroxide gel respectively. The control group was given normal saline injection correspondingly ad. The vaccine specific peptides antibody titers were detected on days 0, 14, 28, 42, 56 and 70 by ELISA. On day 70, the vaccine group was treated with statin, simvastatin (10 mg/kg/day), by intragastrical administration lasting for 4 weeks. The blood pressure of animals was measured using the tail-cuff method (BP-98A, Softron, Japan). All mice were sacrificed on day 98. For safety consideration, the kidney histological changes were observed through HE, Masson and PAS staining. The renal function including sera creatinine and urea nitrogen was detected by biochemical test.

### Sera lipids determination

Before the measurement, all of the mice were fasted for 12 hours. The sera were separated by centrifugation at a rate of 1,000 g for 10 min at room temperature. The sera lipids including TC, TG, HDL-C and LDL-C were measured using biochemical kits.

### Plasma PCSK9 quantification

Plasma PCSK9 level was tested by a mouse PCSK9 ELISA kit (R&D Systems) by comparing experimental sera samples to an internal standard curve according to manufacturer’s protocol. PCSK9 level was quantified both before and after removal of immunoglobulin using Protein G-coated magnetic beads (Life Technologies). Plasma samples were incubated for 10 min with magnetic Protein G beads at room temperature. The Protein G beads were isolated using a magnet, and the immunoglobin-free supernatant was then used directly in PCSK9 ELISA kit.

### LDLR, SREBP-2, HNF-1α, and HMG-CoA reductase expression in liver

Total RNA was extracted from liver tissue by using the TRIzol Reagent (Invitrogen) following the manufacturer’s protocol. The cDNA was synthesized from 20 μl reverse transcribing reaction (Takara) by using 1 μg total RNA. The expression of the associated genes was assessed using qRT-PCR performed at Step One Real-Time PCR machine (Applied Biosystems) using Platinum SYBR qPCR superMix-UDG (Invitrogen). Primers were showed in supplementary Table S2. The expression level of LDLR in liver was also detected by western blotting. The β-actin expression was used as the reference control. The primary antibodies are LDL Receptor (1:1000, Abcam) and β-actin (1:1000, Cell Signaling Technology).

### Statistical analysis

Data are expressed as means ± standard error of the mean (SEM). Student’s t-test was used for comparisons between two groups and a one-way analysis of variance was used for comparisons of three or more groups. The calculations were performed using SPSS (version18.0.0) statistical software. p < 0.05 was accepted as significant.

### Data Availability

All data generated or analysed during this study are included in this published article (and its Supplementary Information files).

## Electronic supplementary material

Supplementary Information
