# Supplementary material for: A Therapeutic Peptide Vaccine Against PCSK9
Source: Sci Rep. 2017 Oct 2;7:12534. doi: 10.1038/s41598-017-13069-w (PMC5624949; doi:10.1038/s41598-017-13069-w)

A Therapeutic Peptide Vaccine Against PCSK9

Yajie Pan[1,2,3+], Yanzhao Zhou[1,2,3+], Hailang Wu[1,2,3], Xiao Chen[1,2,3], Xiajun Hu[1,2,3], Hongrong Zhang[1,2,3], Zihua Zhou[1,2,3], Zhihua Qiu[1,2,3*], Yuhua Liao[1,2,3*]

Table S1 Animal parameters

Day	Parameter	Control	PCSK9Qβ-003
0	SBP(mmHg)	110±3	113±5
	DBP(mmHg)	85±5	84±3
	HR(bpm)	587±12	603±13
	BW(g)	19±2	20±2
14	SBP(mmHg)	115±4	114±2
	DBP(mmHg)	88±2	86±2
	HR(bpm)	598±11	611±20
	BW(g)	23±3	23±2
28	SBP(mmHg)	116±4	115±5
	DBP(mmHg)	90±3	87±5
	HR(bpm)	605±12	594±14
	BW(g)	25±2	25±2
42	SBP(mmHg)	118±6	116±4
	DBP(mmHg)	84±5	88±3
	HR(bpm)	615±10	592±13
	BW(g)	26±2	27±3
70	SBP(mmHg)	117±7	118±5
	DBP(mmHg)	87±5	86±5
	HR(bpm)	610±13	602±15
	BW(g)	31±2	30±3

SBP: systolic blood pressure; DBP: diastolic blood pressure; HR: heart rate; BW: body weight.

Table S2 Primers sequence for quantitative real-time PCR

**Molecules**		**Sequence (5'-3')**
β-actin	forward	CGTTGACATCCGTAAAGACCTC
	reverse	TAGGAGCCAGGGCAGTAATCT
LDL-R	forward	CCATCTTCTTCCCTATTGC
	reverse	GCTCGTCCTCTGTGGTCTT
SREBP-2	forward	TGGCTGGTAAATGGTGTGA
	reverse	AAGCAGGTTTGTAGGTTGG
HNF-1α	forward	AAAAAACCCCAGCAAGGAA
	reverse	GGCAAACCAGTTGTAGACA
HMG-CoA reductase	forward	GGACCAACCTTCTACCTCA
	reverse	ACAGTGCCACATACAATTC

Supplementary Figure 1

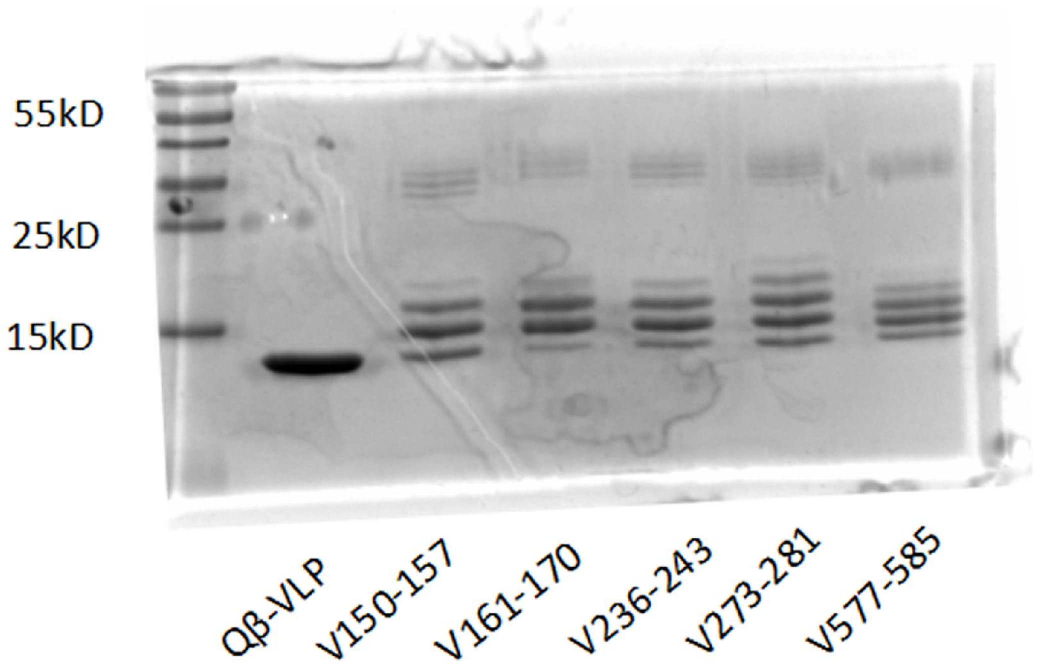

Supplementary Figure 2

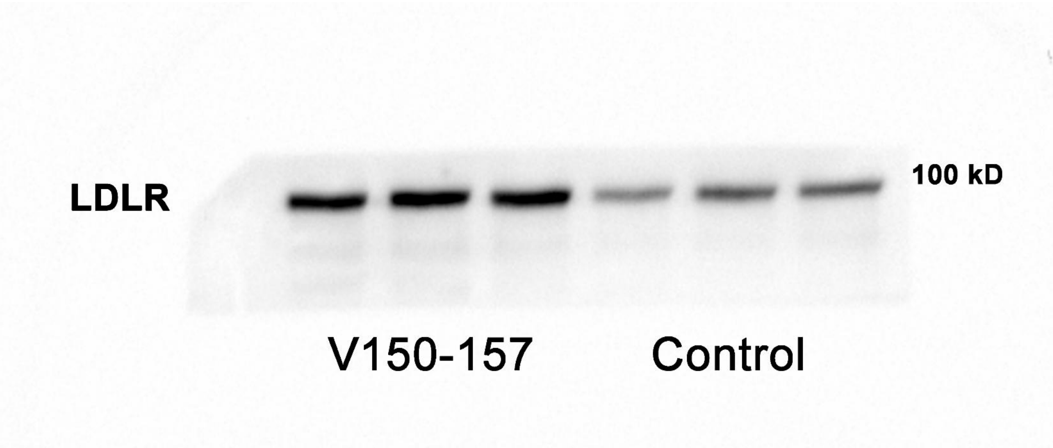

Supplementary Figure 3

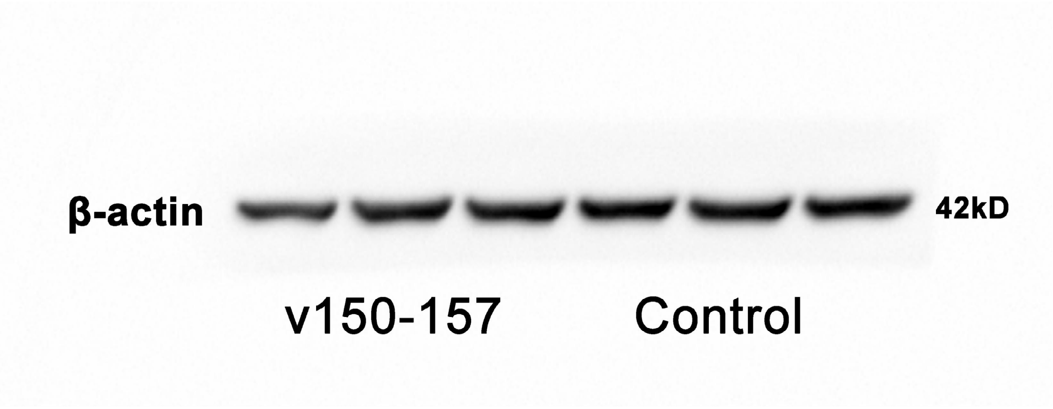

Supplement: Supplementary file 1 — Supplementary Information [file 41598_2017_13069_MOESM1_ESM.pdf]
